# circSCAF11 Attenuates TGF-β/SMAD2/3 Signaling to Suppress Colorectal Cancer Progression

**DOI:** 10.3390/cancers18193128

**Published:** 2026-09-27

**Authors:** Ling He, Bin Liu, Zhaoyong Li

**Affiliations:** Hunan Research Center of the Basic Discipline for Cell Signaling, College of Biology, Hunan University, Changsha 410082, China

**Keywords:** colorectal cancer, circSCAF11, TGF-β signaling pathway, EMT, apoptosis

## Abstract

Circular RNAs with high stability offer potential advantages for developing therapeutic agents against cancers. CircSCAF11 has been reported to be downregulated in CRC, yet its functional mechanisms remain largely unknown. In this study, we demonstrate that circSCAF11 is a genuine circular RNA generated by back-splicing of exon 5 of the *SCAF11* pre-mRNA. We further show that ectopic expression of circSCAF11 inhibits CRC cell proliferation and colony formation. Moreover, its overexpression suppresses the intracellular TGF-β/Smad2/3 signaling pathway and attenuates the epithelial–mesenchymal transition (EMT), migration, and invasion activated by this pathway, while promoting cell apoptosis through the same pathway. In vivo experiments further confirmed that circSCAF11 suppresses CRC tumor growth in nude mouse xenograft models. Collectively, our findings provide novel theoretical insights into the development of circSCAF11-based therapeutic strategies for CRC.

## Simple Summary

Circular RNAs (circRNAs) are evolutionarily conserved, covalently closed-loop RNAs with greater stability than linear transcripts. Their differential expression between tumor and adjacent non-malignant tissues, together with their involvement in multiple hallmarks of tumor progression, positions circRNAs as ideal candidates for cancer diagnostic and prognostic biomarkers and as promising therapeutic agents. CircSCAF11 levels are reduced in colorectal cancer (CRC), but how it works remains unclear. Here, we show that circSCAF11 is a genuine circRNA formed by back-splicing of exon 5 of the SCAF11 pre-mRNA. Restoring circSCAF11 in CRC cells slowed their growth and colony formation. It also suppressed the TGF-β/Smad2/3 signaling pathway, weakening epithelial–mesenchymal transition (EMT), migration, and invasion, while causing more cancer cells to die. In mice, circSCAF11 suppressed tumor growth. Because circRNAs are stable and can be synthesized, circSCAF11 may serve as a new treatment option for advanced CRC.

## 1. Introduction

Colorectal cancer (CRC) is the third most commonly diagnosed cancer and the second leading cause of cancer-related deaths worldwide [1]. Although early screening and diagnosis now enable timely intervention for many individuals, CRC remains a significant global health burden [2,3,4]. Approximately 50% of CRC patients develop metastasis, which poses daunting therapeutic challenges and serves as a major contributor to cancer-associated mortality [5,6]. EMT, which confers CRC cells the ability to migrate from the primary tumor to distant organs during the initial phases of metastasis, promotes tumor dissemination and metastasis when aberrantly activated in cancer [7,8]. In CRC, EMT-associated metastasis is regulated by multiple signaling pathways, including the TGF-β signaling pathway [9]. Additionally, TGF-β signaling is involved in various other cellular processes, such as growth, apoptosis, angiogenesis, and immune responses [10,11]. Therefore, targeting the TGF-β pathway may offer a promising therapeutic strategy for the treatment of CRC.

Circular RNAs (circRNAs) are a common class of specialized RNAs, generally classified into four categories: exonic circRNAs (ecircRNAs), exon-intron circRNAs (EIciRNAs), intronic circRNAs (ciRNAs), and mitochondria-encoded circRNAs (mecciRNAs) [12]. CircRNAs not only typically function as a class of endogenous non-coding RNAs but can also be translated via cap-independent mechanisms to produce bioactive peptides or proteins [13]. Owing to their aberrant expression in cancer and their potential for diagnosis and treatment, a growing body of research has focused on advancing circRNA-based therapies toward clinical application [14,15,16]. Accumulating evidence has shown that circRNAs exhibit diverse biological functions in CRC cells, including acting as microRNA sponges, interacting with proteins, regulating transcription, and participating in signal transduction [17,18]. Consequently, these molecules regulate a variety of cellular processes, including proliferation, angiogenesis, apoptosis, and EMT, thereby positioning circRNAs as potential therapeutic targets in CRC.

In this study, we identified circSCAF11 as a circular RNA localized in both the cytoplasm and nucleus of CRC cells. Overexpression of circSCAF11, which does not affect the mRNA expression of its parental gene SCAF11, suppresses CRC cell proliferation and colony formation. Moreover, we demonstrate that circSCAF11 inhibits EMT, migration, and invasion in CRC cells. Additionally, circSCAF11 promotes apoptosis in CRC cells. Mechanistically, the tumor-suppressive function of circSCAF11 is primarily attributed to its ability to suppress the TGF-β signaling pathway, which has been shown to activate EMT, migration, and invasion while inhibiting apoptosis in CRC [9,10,11,19]. Furthermore, xenograft tumor experiments in nude mice confirmed that circSCAF11 represses in vivo tumor growth of CRC by suppressing the TGF-β signaling pathway. Therefore, circSCAF11 may serve as a novel circular RNA molecular target to guide clinical treatment of CRC.

## 2. Materials and Methods

### 2.1. Cell Lines and Cell Culture

Human CRC cell lines HCT116 (#CL-0096) and HT-29 (#CL-0118), as well as human embryonic kidney HEK293T cells (#CL-0005), were all obtained from Procell (Wuhan, China). These cells were cultured in Dulbecco’s Modified Eagle’s Medium (DMEM) (Gibco, Grand Island, NY, USA) supplemented with 10% fetal bovine serum (FBS), 100 U/mL penicillin, and 100 μg/mL streptomycin. All cultures were kept at 37 °C in a humidified incubator with 5% CO_2_.

### 2.2. RNA Isolation, RT-PCR, and RT-qPCR

Total RNA was extracted from tissues and cell lines using TRIzol™ reagent (Thermo Fisher Scientific, Carlsbad, CA, USA). One microgram of total RNA was reverse-transcribed into cDNA using HiScript II Q RT SuperMix (+gDNA wiper) (#R223, Vazyme, Nanjing, Jiangsu, China) for PCR or quantitative real-time PCR (qPCR). qPCR was performed on a Bio-Rad (Hercules, CA, USA) CFX96 TOUCH system using SYBR^®^ Green I (#Q711, Vazyme). CircRNA and mRNA levels were normalized to β-actin and calculated using the 2^−ΔΔCT^ method. Primer sequences are listed in Tables S1 and S2.

### 2.3. Cytoplasmic/Nuclear Fraction Assay

CRC cells (1 × 10^7^) were resuspended in 200 μL of lysis buffer composed of 10 mM Tris (pH 8.0), 140 mM NaCl, 1.5 mM MgCl_2_, and 0.5% NP-40, followed by incubation on ice for 10 min [18]. For protein detection, 10 μL of lysate was taken as Input; for RNA detection, 10 μL of lysate was added to 1 mL of TRIzol for RNA extraction and used as Input. The remaining lysate was then centrifuged at 15,000× *g* for 10 min at 4 °C. The supernatant was carefully collected as the cytoplasmic fraction. Subsequently, the remaining cell pellet was washed three times with 500 μL of lysis buffer. After the final wash, the supernatant was thoroughly aspirated and discarded, and the pellet was further processed as the nuclear fraction. For protein analysis, an appropriate amount of protein lysis buffer (50 mM Tris-Cl [pH 7.4], 150 mM NaCl, 1% NP-40, 5 mM EDTA, 5 mM EGTA, and 5% glycerol) was added to the pellet, and after thorough lysis and centrifugation, the supernatant was collected as the nuclear protein fraction. GAPDH (#60004-1-Ig, Proteintech, Rosemont, IL, USA) and Lamin A/C (#R26947, Zenbio, Chengdu, Sichuan, China) served as cytoplasmic and nuclear loading controls, respectively, for western blot analysis. For RNA analysis, 1 mL TRIzol reagent was directly added to the pellet, and RT-qPCR analysis was performed on the extracted nuclear RNA. GAPDH mRNA and U1 snRNA were used as cytoplasmic and nuclear markers, respectively. Primer sequences are listed in Table S2.

### 2.4. Western Blotting Analysis

Total protein was isolated from tissue samples and cultured cell lines by homogenization in protein lysis buffer supplemented with protease inhibitors. 20 μg of each sample was then resolved by 8% sodium dodecyl sulfate-polyacrylamide gel electrophoresis (SDS-PAGE). The separated proteins were then transferred onto 0.45-μm polyvinylidene fluoride (PVDF) membranes (Amersham, GE Healthcare, Piscataway, NJ, USA), followed by blocking for 1 h with 5% skimmed milk prepared in TBST buffer. Subsequently, the membranes were probed overnight at 4 °C with specific primary antibodies against E-cadherin (1:2000, #60335-1-Ig, Proteintech), N-cadherin (1:2000, #22018-1-AP, #66219-1-Ig, Proteintech), Vimentin (1:10,000, #60330-1-Ig, Proteintech), THBS1 (1:1000, #R27353, Zenbio), Noggin (1:1000, #84283-5-RR, Proteintech), Smad6 (1:1000, #18342-1-AP, Proteintech), full caspase-3 (1:1000, #19677-1-AP, Proteintech), cleaved caspase-3 (1:1000, #9661, cell signaling technology, Danvers, MA, USA), PARP1 (1:1000, #13371-1-AP, Proteintech), Smad2 (1:1000, #200790, Zenbio), Phospho-Smad2 (Ser467) (1:1000, #310079, Zenbio), Smad3 (1:1000, #R25743, Zenbio), Phospho-Smad3 (Ser423/425) (1:1000, #R22919, Zenbio), or β-actin (1:5000, #66009-1-Ig, Proteintech). The membranes were then subjected to three 5-min washes in TBST and probed for 1 h at room temperature with horseradish peroxidase (HRP)-labeled goat anti-mouse (#A0216, Beyotime, Shanghai, China) or goat anti-rabbit (#A0208, Beyotime) secondary antibodies. Protein bands were detected by enhanced chemiluminescence (ECL) western blotting substrate (#P0018FM, Beyotime). Finally, the grayscale intensities of the protein bands were measured with ImageJ software (version 1.52a).

### 2.5. Vector Construction and Plasmid Transfection

To generate the circSCAF11 overexpression construct, a 2718-bp fragment corresponding to exon 5 of human circSCAF11 was amplified by PCR and cloned into the EcoRI/BamHI sites of the lentiviral vector pLC5-ciR (Geenseed Biotech, Guangzhou, China). The primer sequences used are provided in Table S3. For lentivirus production, the lentiviral vectors were co-transfected with the packaging plasmids psPAX2 and pMD2.G into HEK293T cells for 24 or 48 h. All transfection procedures were performed using PEI MAX 40 K (#24765, Polysciences, Warrington, PA, USA) following the manufacturer’s instructions. The lentivirus-containing cell culture supernatant was collected, filtered, and supplemented with 8 μg/mL polybrene. After removal of the original medium, the viral supernatant was added to the culture dish to infect HCT116 and HT-29 cells. Stable cell lines were established through selection with 1.5 μg/mL puromycin (#HY-K1057, MCE, Monmouth Junction, NJ, USA) over a period of 2 weeks.

### 2.6. Cell Proliferation Assay

Cell proliferation was assessed using a cell-counting assay. CRC cells infected with lentivirus for 48 h were seeded into 12-well plates (5 × 10^4^ cells/well, in triplicate) and cultured for 24, 48, 72, or 96 h. At each time point, cells were stained with 0.4% trypan blue, and viable cells were counted with a hemocytometer.

### 2.7. Colony Formation Assay

CRC cells infected with lentivirus for 48 h were seeded into 6-well plates (1 × 10^3^/well) and cultured for 10–14 days. Colonies were fixed with 4% paraformaldehyde (15 min), stained with 0.5% crystal violet (20 min), and washed with PBS. Images were captured and analyzed using ImageJ (version 1.52a). Plating efficiency was calculated as the ratio of the number of colonies formed to the number of cells seeded.

### 2.8. RNA-Seq

Total RNA was isolated from HCT116 cells stably expressing the empty vector pLC5-ciR (EV) or circSCAF11 (three biological replicates per group) using TRIzol™ reagent (Thermo Fisher Scientific). Qualified RNA samples were enriched with oligo(dT) magnetic beads and used to construct strand-specific cDNA libraries with the NEBNext Ultra II RNA Library Prep Kit for Illumina (NEB, Ipswich, MA, USA). Pooled libraries were sequenced on the Illumina NovaSeq X Plus platform in PE150 mode (Personalbio, Shanghai, China). Raw FASTQ data were filtered by removing 3′ adapter sequences with Fastp (v0.22.0) and discarding reads with average quality < Q20. Clean reads were aligned to the reference genome using HISAT2 (v2.1.0) after index construction. Read counts per gene were quantified with HTSeq (v0.9.1), normalized as FPKM. Differential expression analysis was performed with DESeq2 (v1.38.3). *p* values were adjusted for multiple testing using the Benjamini–Hochberg (BH) method, and genes with |log2FoldChange| > 1 and FDR < 0.05 (i.e., BH-adjusted *p* value) were considered differentially expressed. Enrichment analysis was conducted with clusterProfiler (v4.6.0) to identify significantly enriched KEGG pathways (hypergeometric test, *p* < 0.05). GSEA (clusterProfiler v4.6.0) ranked all genes by differential expression and tested predefined gene sets for enrichment at the ranked list extremes, indicating pathway activation or inhibition without preset thresholds.

### 2.9. Wound Healing Assay

Transfected cells in the logarithmic growth phase were prepared as single-cell suspensions and plated in 6-well plates. Upon reaching 90–100% confluence, cells were pretreated with mitomycin C (10 µg/mL) for 2 h at 37 °C to exclude the confounding effect of cell proliferation on wound closure. A uniform scratch was then generated using a 200-µL pipette tip, and the wells were gently washed three times with PBS to remove detached cells. Cells were subsequently maintained in medium supplemented with 2% FBS at 37 °C. Images were captured at 0 and 24 h post-scratching using an inverted microscope (EVOS FL, Thermo Fisher), and wound area was quantified using ImageJ software.

### 2.10. Transwell Invasion Assay

Transwell invasion assays were performed using 24-well chambers (8.0 μm pore size; BD, Franklin Lakes, NJ, USA) precoated with Matrigel (#356234, Corning, Corning, NY, USA). Transfected cells (1.5 × 10^5^ to 2.5 × 10^5^) were resuspended in serum-free medium and seeded into the upper chamber; the lower chamber contained DMEM with 20% FBS. After 48 h, non-invading cells were removed from the upper surface. Invading cells on the lower surface were fixed with 4% paraformaldehyde (20 min), stained with 0.5% crystal violet (10 min), and washed. Images were captured using an inverted microscope (EVOS M5000, Thermo Fisher), with at least three random fields counted per well.

### 2.11. Flow Cytometry Assay

Apoptosis was assessed using the Annexin V-FITC/PI Apoptosis Detection Kit (A211, Vazyme). HCT116 cells were plated in 6-well plates and infected with EV or circSCAF11 lentivirus, with or without SRI-011381 treatment. Cells (1 × 10^5^–5 × 10^5^) were collected, washed twice with cold PBS, resuspended in 100 μL binding buffer, and stained with 5 μL Annexin V-FITC and 5 μL PI for 10 min at room temperature in the dark. After adding 400 μL binding buffer, samples were immediately analyzed on a flow cytometer (CytoFLEX, Beckman Coulter, Brea, CA, USA). Untreated cells served as negative controls; single-stained cells were used for compensation. Data were analyzed using FlowJo v10.

### 2.12. Animal Experiments

All animal procedures were approved by the Institutional Animal Care and Use Committee of Hunan University. Female BALB/c nude mice (5 weeks old) purchased from Charles River Laboratories (Beijing, China) were housed in a specific pathogen-free environment under a 12 h light/dark cycle. For xenograft models, mice were randomly divided into four groups (*n* = 6 per group) and subcutaneously inoculated with HCT116 EV or HCT116 circSCAF11 stable cells (1.0 × 10^7^ cells) into the dorsal flank. SRI-011381 was prepared as a 50 mg/mL DMSO stock and diluted with PEG300, Tween 80, and saline (final DMSO ≤ 2%). SRI-011381 or vehicle control was administered to tumor-bearing mice at 30 mg/kg via intraperitoneal injection (100–200 μL) once every two days. Tumor volume was measured every three days using the formula V = (L × W^2^)/2. Mice were sacrificed on day 22, and subcutaneous tumors were removed and weighed. Group allocation was not blinded; however, the investigator responsible for allocation was not involved in any subsequent procedures. All other experimental phases, including intraperitoneal injection, outcome measurements (tumor volume and tumor weight), and data analysis, were performed under blinded conditions.

### 2.13. In Vivo Immunofluorescence Staining Analysis

For immunofluorescence staining, isolated tumors were fixed in 4% PFA at 4 °C for 2 h, followed by 30% sucrose at 4 °C overnight. Tumors were then embedded in OCT and frozen at −80 °C. Sections (6 μm) were cut using a freezing microtome (LEICA CM1950). TUNEL staining was performed per the manufacturer’s instructions (C1089, Beyotime). Ki67 staining used a primary anti-Ki67 antibody (#27309-1-AP, Proteintech) overnight at 4 °C, followed by donkey anti-rabbit 555 secondary antibody (A-31572, Invitrogen, Carlsbad, CA, USA). Images were captured using a two-photon laser scanning microscope (Nikon, Melville, NY, USA).

### 2.14. Statistical Analysis

All data are expressed as the mean ± standard deviation (SD) from a minimum of three independent experiments. For imaging-based experiments, at least three randomly selected fields of view were analyzed statistically, and representative images are presented. Differences between two groups were assessed using a two-tailed unpaired or paired Student’s *t*-test, while one-way or two-way analysis of variance (ANOVA) was used for comparisons among three or more groups. All statistical analyses were carried out with Microsoft Excel 2016 and GraphPad Prism 9.0. A *p* value below 0.05 was regarded as statistically significant (ns: not significant; * *p* < 0.05, ** *p* < 0.01, *** *p* < 0.001, **** *p* < 0.0001).

## 3. Results

### 3.1. Identification and Characterization of circSCAF11

According to circBase, circSCAF11 (hsa_circ_0025967) is a 2718-nt circRNA generated by back-splicing of exon 5 of the SCAF11 gene located on human chromosome 12 (Figure 1A). It was identified as one of the most downregulated circRNAs in CRC tissues [20]. The back-splicing of endogenous circSCAF11 was confirmed by PCR using convergent and divergent primers, with β-actin serving as a control for linear and circular RNAs, respectively. As expected, the convergent primers amplified PCR products from both complementary DNA (cDNA) and genomic DNA (gDNA) templates for circSCAF11 and β-actin (Figure 1B). However, when cDNA was used as a template, the divergent primers amplified a PCR product for circSCAF11 but not for β-actin. Sanger sequencing also validated that the back-splicing junction region matched the reported sequence (Figure 1C). Unlike SCAF11 and β-actin mRNAs, which were susceptible to degradation by the exonuclease RNase R, circSCAF11 remained stable following RNase R treatment (Figure 1D). Actinomycin D chase assays also demonstrated that circSCAF11 exhibited a longer half-life compared to linear SCAF11 and β-actin mRNAs (Figure 1E). Cellular fractionation experiments revealed that circSCAF11 was localized in both the nucleus and cytoplasm of HCT116 and HT-29 cells (Figure 1F), indicating that this circRNA may play a role at either the transcriptional or post-transcriptional level in gene expression.

Then, we evaluated whether circSCAF11 has protein-coding potential in the cytoplasm. Some circRNAs can produce functional peptides or biologically active proteins via cap-independent translation mediated by internal ribosome entry sites (IRESs), which directly recruit ribosomes [13,21,22]. Additionally, m6A modification in circRNAs also facilitates the assembly of the translation initiation machinery to drive protein synthesis. Using the IRESite website (http://iresite.org/), we predicted putative IRES elements within the circSCAF11 sequence but found no statistically significant signals. The circSCAF11 sequence was also analyzed using circPrimer2.0 software for the presence of open reading frames (ORFs), IRES, and coding-competent m6A modifications [23]. The results revealed 11 ORFs within the circSCAF11 sequence (Figure S1A), but no IRES elements or high-confidence m6A modifications were predicted (Figure S1B). Therefore, circSCAF11 lacks two key cap-independent translation elements, namely IRES and high-confidence m6A modifications, suggesting a low probability of protein-coding ability.

### 3.2. circSCAF11 Inhibits the Proliferation of CRC Cells

Consequently, we aim to further investigate the possible functions of this circRNA as a non-coding RNA in this study. To explore the potential biological function of circSCAF11 in CRC, we established a circSCAF11 overexpression system, which successfully overexpressed circSCAF11 in HCT116 and HT-29 cells via lentiviral infection (Figure 2A). However, SCAF11 mRNA expression was not affected in CRC cells with high exogenous circSCAF11 expression, indicating that circSCAF11 does not regulate the expression of its parent gene, SCAF11 (Figure 2B,C). To determine whether circSCAF11 functions in an oncogenic or tumor-suppressive capacity, we first assessed its effect on CRC cell proliferation. Cell counting assays revealed that circSCAF11 overexpression led to a markedly reduced cell number (Figure 2D). In addition, colony formation assays demonstrated that overexpression of circSCAF11 decreased the clonogenic survival of CRC cells compared with EV groups (Figure 2E). These findings suggest circSCAF11 may play a tumor-suppressive role in CRC cells.

### 3.3. circSCAF11 Is Functionally Associated with the TGF-β Signaling Pathway in CRC Cells

To investigate the functional role of circSCAF11 in CRC, we generated an HCT116 cell line stably expressing circSCAF11 (Figure 3A). Based on prior evidence that circular RNAs participate in transcriptional regulation in the nucleus [24,25,26], we analyzed the transcriptome of cells with stable circSCAF11 overexpression and EV control. Transcriptome sequencing was performed on these two cell lines, and differentially expressed genes were identified (Figure 3B). Based on the above results, pathway enrichment analysis of the differentially expressed genes was conducted using the KEGG database (Figure 3C). The analysis revealed that the differentially expressed genes in HCT116 cells with circSCAF11 overexpression were primarily enriched in multiple signaling pathways closely associated with malignant tumor progression, including the p53 signaling pathway, Hippo signaling pathway, Wnt signaling pathway, mTOR signaling pathway, and TGF-β signaling pathway. Furthermore, KEGG-based gene set enrichment analysis (GSEA) demonstrated that the TGF-β signaling pathway gene set was positively enriched in circSCAF11-overexpressing CRC cells (Figure 3D). qPCR assay confirmed that circSCAF11 overexpression significantly upregulated the mRNA levels of TGF-β pathway-related genes, including FBN1, Noggin, and Smad6, while it downregulated THBS1 mRNA (Figure 3E). Consistent with the mRNA assay, western blot analysis showed that the increase in Noggin and SMAD6 proteins was accompanied by a decrease in THBS1 protein in HCT116 cells with circSCAF11 transfection (Figure 3F). Taken together, these observations suggest that circSCAF11 may be functionally linked to the TGF-β signaling pathway in CRC cells.

### 3.4. circSCAF11 Reverses TGF-β/Smad2/3-Induced EMT, Migration, and Invasion in CRC Cells

TGF-β can drive EMT in multiple tumor types, facilitating metastasis and conferring therapy resistance [27]. To investigate whether circSCAF11 regulates TGF-β pathway-mediated EMT, we analyzed the protein levels of EMT markers in HCT116 and HT-29 cells with circSCAF11 lentiviral transduction plus a TGF-β1 signaling agonist, SRI-011381 (SRI), or not. The results show that the levels of phosphorylated Smad2 (p-Smad2) and phosphorylated Smad3 (p-Smad3) were markedly increased in the SRI-treated group, while total Smad2 and Smad3 protein levels remained unchanged compared to the EV control group (Figure 4A,B). Importantly, circSCAF11 overexpression blocked the SRI-induced upregulation of p-Smad2 and p-Smad3 protein levels in CRC cells. The results also demonstrated that, compared to the EV group, the protein abundance of the epithelial marker E-cadherin was significantly upregulated in the circSCAF11-overexpressing group, whereas the mesenchymal markers N-cadherin and Vimentin showed a downward trend, a molecular phenotype consistent with EMT inhibition. In contrast, the EV + SRI group exhibited decreased E-cadherin protein levels and increased trends in N-cadherin and Vimentin, confirming that activation of the TGF-β pathway by SRI promotes EMT. Notably, circSCAF11 overexpression prevented the SRI-induced changes in the protein expression of the aforementioned EMT markers (Figure 4A,B). Collectively, these findings indicate that circSCAF11 overexpression reverses the EMT phenotype in CRC cells induced by TGF-β signaling activation.

Previous studies have demonstrated that activation of the TGF-β/Smad-mediated EMT signaling pathway enhances tumor cell motility and invasion in CRC [28]. Our wound healing assay revealed that circSCAF11 overexpression inhibited cell migration, whereas the activation of the TGF-β pathway by SRI promoted cell migration (Figure 4C,D). However, the wound healing ability in the circSCAF11 + SRI group was lower than that of the EV + SRI group, suggesting that circSCAF11 overexpression effectively suppressed the enhanced cell migration induced by SRI treatment. We also evaluated the invasive capacity of equal numbers of viable cells in each group using the Transwell invasion assay. CircSCAF11 overexpression was found to inhibit cell invasion (Figure 4E,F). In contrast, SRI, which mediates the activation of the TGF-β pathway, promoted cell invasion. Furthermore, the number of penetrating cells was lower in the circSCAF11 + SRI group than in the EV + SRI group, indicating that circSCAF11 overexpression reversed the SRI-induced increase in invasive capacity. In summary, circSCAF11 suppresses cell migration and invasion, which may be enhanced by the TGF-β signaling pathway via EMT.

### 3.5. circSCAF11 Induces CRC Cell Apoptosis via TGF-β Pathway Inhibition

Apoptosis represents a critical pathway in the pathogenesis of multiple diseases, including CRC. During this process, pro-caspases are proteolytically cleaved into their active forms, exemplified by cleaved caspase-3, which serves as a central executioner of apoptosis. As a well-established substrate of activated caspase-3, PARP1 is inactivated upon cleavage, thereby suppressing DNA damage repair and promoting caspase-mediated DNA fragmentation [29]. Previous studies have reported that activation of the TGF-β pathway inhibits apoptosis, whereas its inhibition induces apoptosis in CRC cells [30,31]. Western blot analysis showed that, compared to the EV control group, the protein levels of cleaved caspase-3 and cleaved PARP1 were increased in HCT116 and HT-29 cells with lentivirus-mediated overexpression of circSCAF11 (Figure 5A,B). In contrast, treatment with SRI, an activator of the TGF-β/Smad2/3 signaling pathway, led to downregulation of cellular cleaved caspase-3 and cleaved PARP1 protein levels. However, circSCAF11 overexpression restored the SRI-induced downregulation of these proteins in CRC cells, indicating that circSCAF11 blocks the inhibitory effect of the TGF-β signaling pathway on these apoptosis-executing proteins. Additionally, compared with the EV control group, circSCAF11 overexpression increased the apoptosis rate in CRC cells, whereas SRI treatment decreased the apoptosis rate (Figure 5C,D). Furthermore, circSCAF11 significantly reversed the SRI-induced suppression of apoptosis, as shown by the higher apoptotic rate in the circSCAF11 + SRI group versus the EV + SRI group. Taken together, our data suggest that circSCAF11 facilitates CRC cell apoptosis, possibly via negative regulation of TGF-β signaling.

### 3.6. circSCAF11 Inhibited CRC Tumor Growth In Vivo

Inhibition of proliferation, as well as induction of apoptosis, is closely associated with tumor regression. Cell proliferation assays showed that, compared to the EV group, overexpression of circSCAF11 significantly reduced the cell growth rate (Figure 6A,B). Moreover, ectopic expression of circSCAF11 inhibited the enhancing effect of SRI on cell proliferation. Next, we examined the effect of circSCAF11 on CRC growth in vivo using a xenograft mouse model. We subcutaneously injected HCT116 cells from the EV or circSCAF11-overexpression groups into BALB/c nude mice. Consistent with the in vitro results, the growth rate of circSCAF11-expressing xenograft tumors was significantly slower than that of the EV group (Figure 6C). Similarly, on average, circSCAF11-expressing mice had smaller tumor volumes and lower tumor weights than the EV group (Figure 6D–F). Western blot analysis revealed that, in mice treated with SRI via intraperitoneal injection, circSCAF11 overexpression impeded the SRI-induced increase in p-Smad2 and p-Smad3 protein levels (Figure 6G), confirming that circSCAF11 inhibits the TGF-β signaling pathway in vivo.

We further examined proliferation and apoptosis in tumor sections. The expression of the cell proliferation marker Ki67 was lower in the circSCAF11 group than in the EV group (Figure 6H). Conversely, TUNEL staining revealed more apoptotic cells in the circSCAF11 group than in the EV group (Figure S2). However, in mice that received an intraperitoneal injection of SRI, the growth rate, tumor volume, and tumor weight were lower in the circSCAF11-overexpression group than in the EV group (Figure 6C–F). Moreover, under SRI treatment, the expression of Ki67 was lower, and the TUNEL signal was higher in the circSCAF11-expressing group than in the EV group (Figure 6H and Figure S2). In summary, our findings suggest that circSCAF11 inhibits tumor growth and promotes apoptosis, as well as antagonizes the TGF-β signaling pathway, in CRC in vivo.

## 4. Discussion

CircRNAs are a novel class of evolutionarily conserved RNAs that form closed continuous loops via covalently linked 3′ and 5′ ends. Owing to their circular configuration, circRNAs exhibit greater stability than linear transcripts. Furthermore, circRNAs show differential expression profiles between tumor tissues and adjacent non-malignant tissues and play crucial roles in several hallmarks of tumor progression. Consequently, these characteristics have positioned circRNAs as ideal candidates for cancer diagnostic and prognostic biomarkers, as well as for the application of engineered circRNAs in clinical cancer therapy [14,15,16,32]. In this study, based on the findings that circSCAF11 promotes apoptosis while inhibiting proliferation, EMT, migration, invasion, and tumor growth in CRC, we identified circSCAF11 as a suppressor of CRC development. We further demonstrated that circSCAF11 suppresses CRC progression by inhibiting the TGF-β/Smad signaling pathway, suggesting its potential as a promising molecular therapeutic agent for the treatment of CRC [33].

TGF-β receptors initiate signaling through two primary pathways: the canonical pathway, which involves Smad proteins, and non-canonical pathways that use alternative signaling mechanisms [34]. In CRC, the TGF-β canonical pathway plays a dual role. In the early stages of cancer development, the TGF-β pathway exerts a suppressive effect. However, as the disease progresses, TGF-β shifts from inhibiting tumor growth to initiating cancer-promoting processes. TGF-β signaling is altered in CRC and contributes to its malignant progression by promoting invasion and metastasis, affecting apoptosis, and regulating immunological responses [9,10,11]. Moreover, EMT is also one of the key mechanisms through which TGF-β drives cancer progression, including CRC [19,27,28]. The TGF-β signaling pathway is widely recognized as a promising therapeutic target in cancer treatment, and various agents—including antibodies, vaccines, and kinase inhibitors—have been developed for cancer therapy [34,35,36]. However, to date, no drugs targeting this pathway have been approved for clinical use.

Various classes of non-coding RNAs (ncRNAs), including miRNAs, lncRNAs, and circRNAs, modulate TGF-β signaling and thus govern the delicate balance between tumor suppression and promotion in CRC [30,37,38]. For instance, through their effect on the TGF-β signaling pathway, certain circRNAs promote, whereas others suppress, the development and progression of CRC [39,40,41]. Our study revealed that circSCAF11 suppresses the TGF-β signaling pathway in CRC cells, partly by downregulating THBS1 expression, a gene known to contribute to CRC metastasis [42,43]. Furthermore, circSCAF11 upregulates Noggin expression, which in turn inhibits CRC progression by suppressing the BMP signaling pathway [44,45]. Together with the finding that circSCAF11 inhibits SRI-enhanced CRC growth in nude mouse xenografts, we propose that this circRNA functions as a potent suppressor of the TGF-β signaling pathway. Given the high stability and synthetic accessibility of circRNAs, circSCAF11, identified in our study, represents a promising TGF-β inhibitor for the therapy of advanced CRC [33].

Nonetheless, several issues in our study remain to be elucidated. For instance, we did not explore the molecular mechanism through which circSCAF11 regulates the transcription of THBS1, Noggin, and SMAD6. Does circSCAF11 influence the transcriptional activity of the aforementioned genes through interactions with RNA polymerase II? Alternatively, does it act as a molecular scaffold to recruit specific transcription factors to gene promoter regions, thereby regulating their expression? Additionally, circSCAF11 is also localized in the cytoplasm but lacks protein-coding potential. Does it exert its functions in the cytoplasm through other mechanisms? Moreover, does circSCAF11 expression correlate with CRC stage, metastatic status, or patient prognosis? These questions represent key directions for future research that require further clarification. Collectively, elucidation of the aforementioned issues will lay a robust groundwork for harnessing circSCAF11 as a future therapeutic agent against CRC.

## 5. Conclusions

In conclusion, we confirm that circSCAF11 is a bona fide circular RNA that functions as a tumor suppressor in CRC. Its overexpression inhibits malignant phenotypes by suppressing the TGF-β/Smad2/3 pathway, thereby blocking EMT, migration, and invasion while promoting apoptosis. These anti-tumor effects were validated in vivo using mouse xenograft models. Collectively, our findings establish circSCAF11 as a promising therapeutic target for CRC intervention.

## Figures and Tables

**Figure 1:**
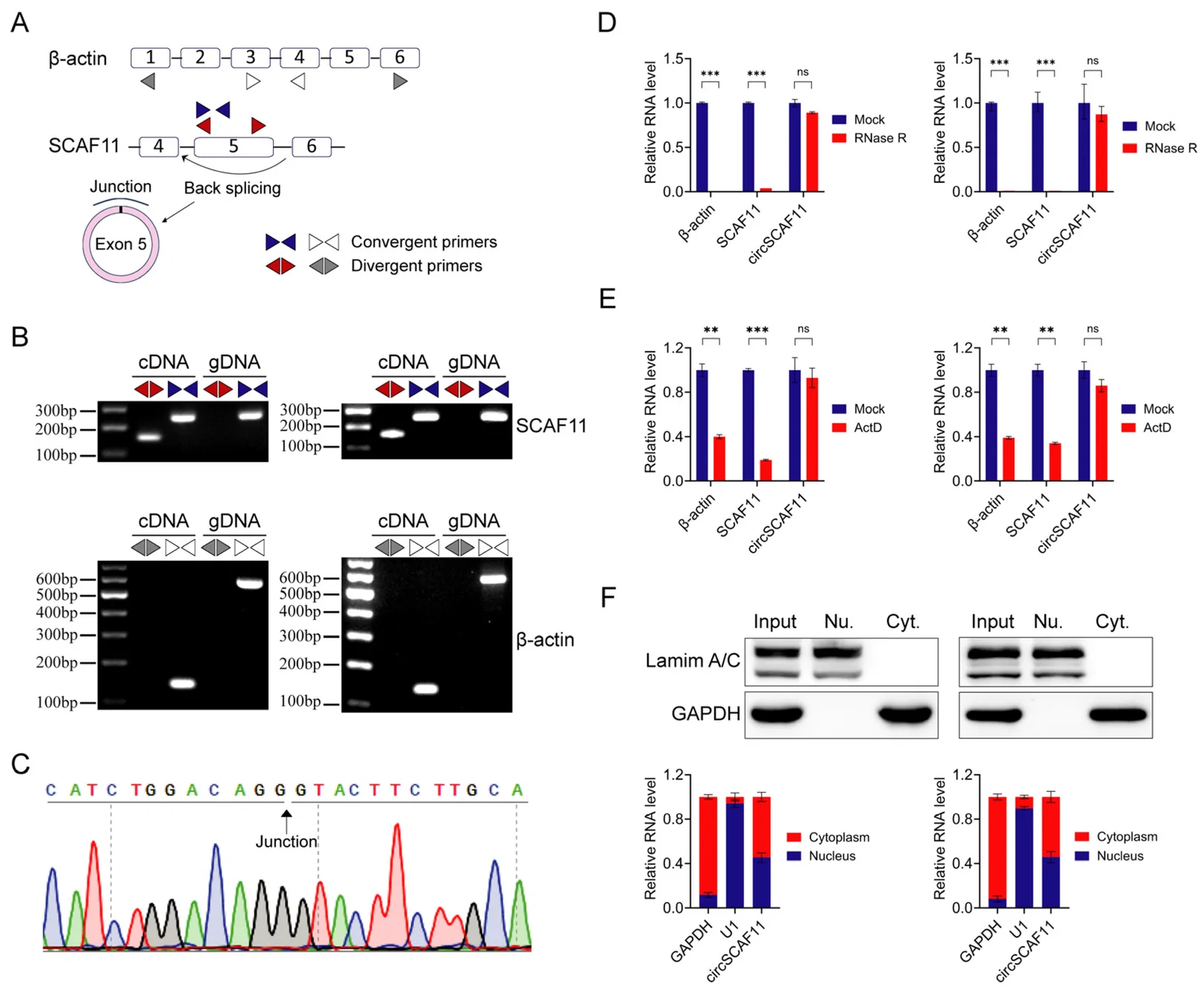
Identification and characterization of circSCAF11. (**A**). Schematic illustration showing the genomic location of circSCAF11 derived from its parent gene, SCAF11. (**B**). Convergent and divergent primers, indicated by triangles of different colors in (**A**), were designed to amplify circSCAF11 or β-actin mRNA by PCR using cDNA and gDNA templates from HCT116 (left) and HT-29 (right) cells, respectively. (**C**). The circSCAF11 junction sequence was confirmed by Sanger sequencing. (**D**). RT-qPCR analysis of β-actin, SCAF11 mRNA, and circSCAF11 expression levels in HCT116 (left) and HT-29 (right) cells treated with 10 U/μg RNase R at 37 °C for 30 min. (**E**). RT-qPCR analysis of β-actin, SCAF11 mRNA, and circSCAF11 expression levels in HCT116 (left) and HT-29 (right) cells treated with 5 μg/mL actinomycin D (Act D) for 24 h. (**F**). The distribution of circSCAF11 in nuclear and cytoplasmic fractions of HCT116 (left) and HT-29 (right) cells was determined by RT- qPCR. U1 snRNA and GAPDH mRNA were used as positive controls for the nucleus and cytoplasm, respectively. Western blotting using antibodies against Lamin A/C and GAPDH confirmed successful nuclear and cytoplasmic fractionation, respectively. Nu., Nucleus; Cyt., Cytoplasm. The whole blots (uncropped blots) are shown in the Supplementary File. ns: not significant; ** *p* < 0.01, *** *p* < 0.001.

**Figure 2:**
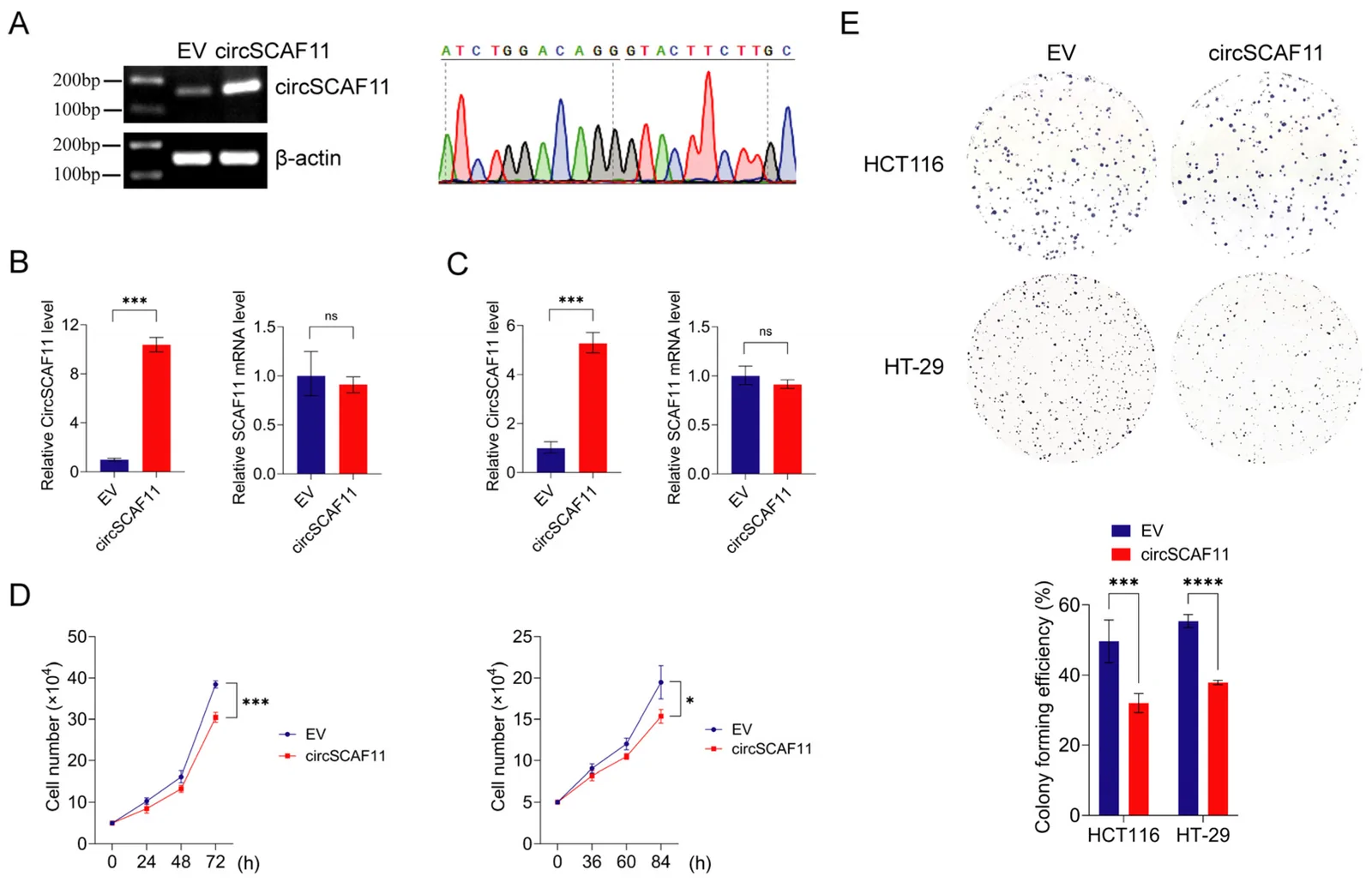
CircSCAF11 inhibits CRC cell proliferation. (**A**). CircSCAF11 overexpression in HCT116 cells was confirmed by RT-PCR and agarose gel electrophoresis, with β-actin as an internal control. The empty vector (EV) was used as a negative control. Sanger sequencing results confirmed the circularization of circSCAF11 derived from the circSCAF11 overexpression plasmid. (**B**,**C**). RT-qPCR analysis of circSCAF11 and SCAF11 mRNA expression levels in HCT116 (**B**) and HT-29 cells (**C**) following circSCAF11 overexpression, with β-actin as a normalization control. (**D**). Cell proliferation was measured by trypan blue counting in HCT116 (left) and HT-29 (right) cells with circSCAF11 overexpression. (**E**). Colony formation assays based on crystal violet staining of colonies detected the cell proliferation of HCT116 and HT-29 cells overexpressing either EV or circSCAF11 after 14 days of culture. ns: not significant; * *p* < 0.05, *** *p* < 0.001, **** *p* < 0.0001.

**Figure 3:**
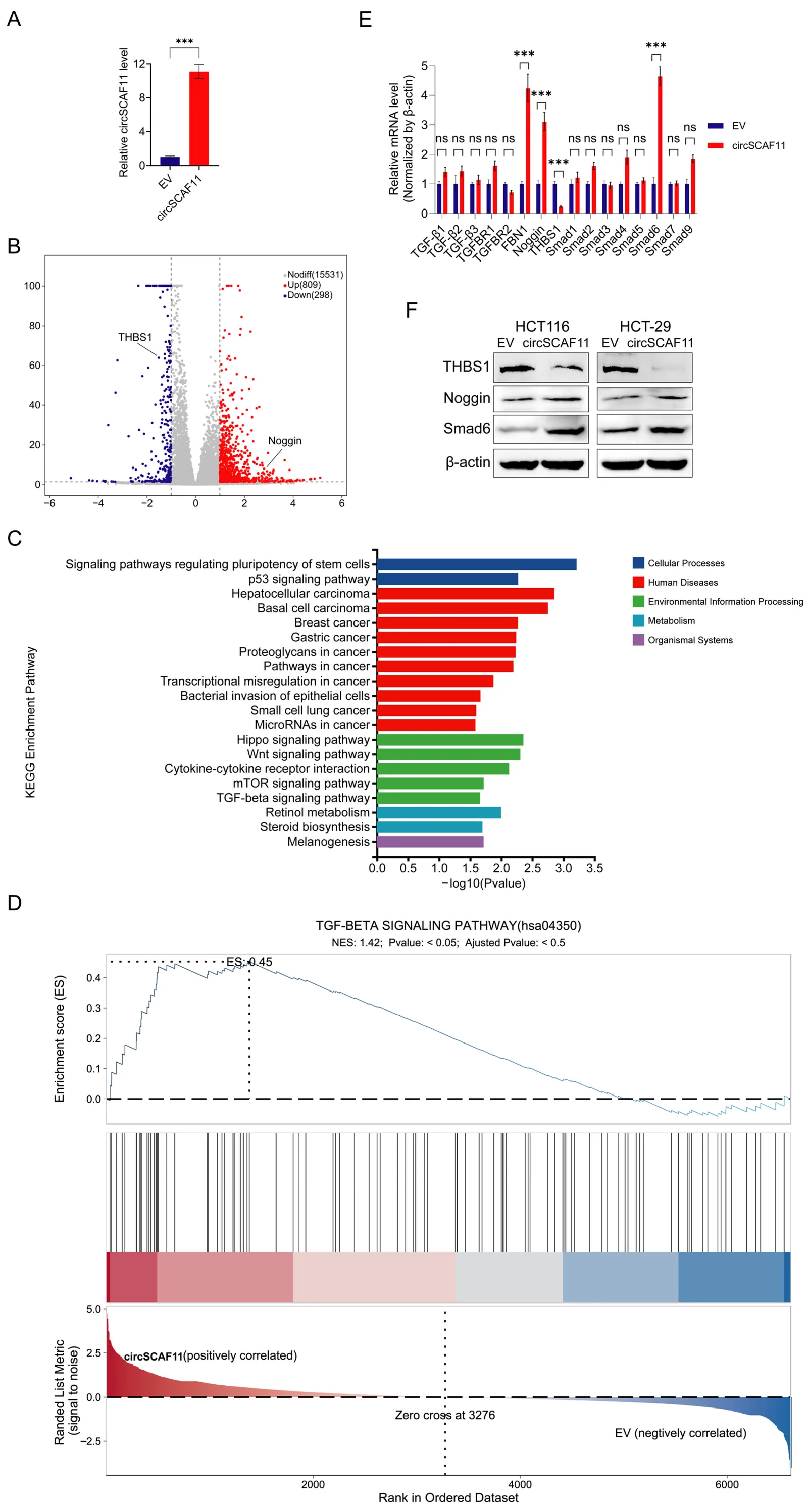
circSCAF11 is functionally linked to the TGF-β signaling pathway in CRC cells. (**A**). RT-qPCR measured circSCAF11 expression levels in stably transfected HCT116 cell lines. (**B**). Volcano plot of differentially expressed genes (DEGs). The x-axis represents the fold change in gene expression (log_2_FC), and the y-axis represents the statistical significance (−log_10_ *p*-value). Based on their expression changes, genes are classified into three categories: significantly upregulated (red dots), significantly downregulated (blue dots), and genes without significant differential expression (gray dots). (**C**). Bar chart showing KEGG pathway enrichment analysis of differentially expressed genes. The chart presents the signaling pathways significantly enriched among DEGs following circSCAF11 overexpression. Enrichment significance is represented as −log_10_(*p*-value). (**D**). KEGG-GSEA plot showing enrichment of the TGF-β signaling pathway gene set in the circSCAF11 overexpression group. (**E**). RT-qPCR analysis of the mRNA expression levels of key molecules in the TGF-β signaling pathway in HCT116 cells stably overexpressing circSCAF11, with β-actin as a normalization control. (**F**). Western blot analysis of the protein expression levels of THBS1, Noggin, and Smad6 in HCT116 and HT-29 cells overexpressing circSCAF11. β-actin was used as a loading control. The whole blots (uncropped blots) are shown in the Supplementary File. ns: not significant; *** *p* < 0.001.

**Figure 4:**
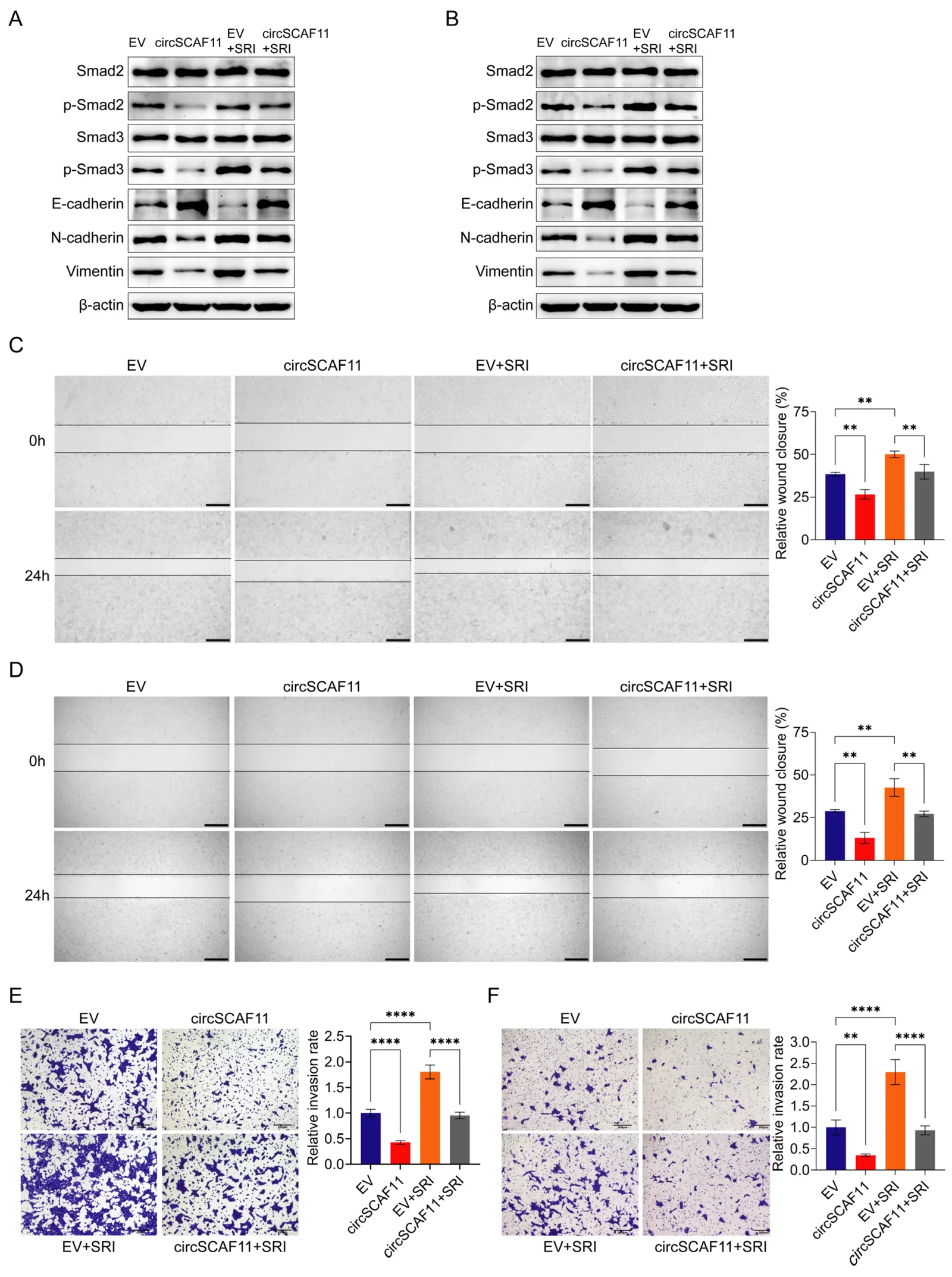
circSCAF11 overexpression reverses TGF-β/Smad2/3-induced EMT, migration, and invasion in CRC cells. (**A**,**B**). The expression levels of Smad2, p-Smad2, Smad3, p-Smad3, E-cadherin, N-cadherin, and Vimentin in HCT116 (**A**) and HT-29 (**B**) cells under the indicated conditions were assessed by Western blot. β-actin was used as a loading control. (**C**,**D**). Wound healing assays were performed to assess the migration ability of HCT116 (**C**) and HT-29 (**D**) CRC cells. Wound closure was calculated as (0 h area − 24 h area)/0 h area × 100% for each experimental condition. Scale bar: 500 μm. (**E**,**F**). Transwell invasion assays were used to determine the invasiveness of HCT116 (**E**) and HT-29 (**F**) CRC cells. Left panels: representative images of crystal violet staining; right panels: quantitative analysis of invasion. Scale bar: 200 μm. The whole blots (uncropped blots) are shown in the Supplementary File. ** *p* < 0.01, **** *p* < 0.0001.

**Figure 5:**
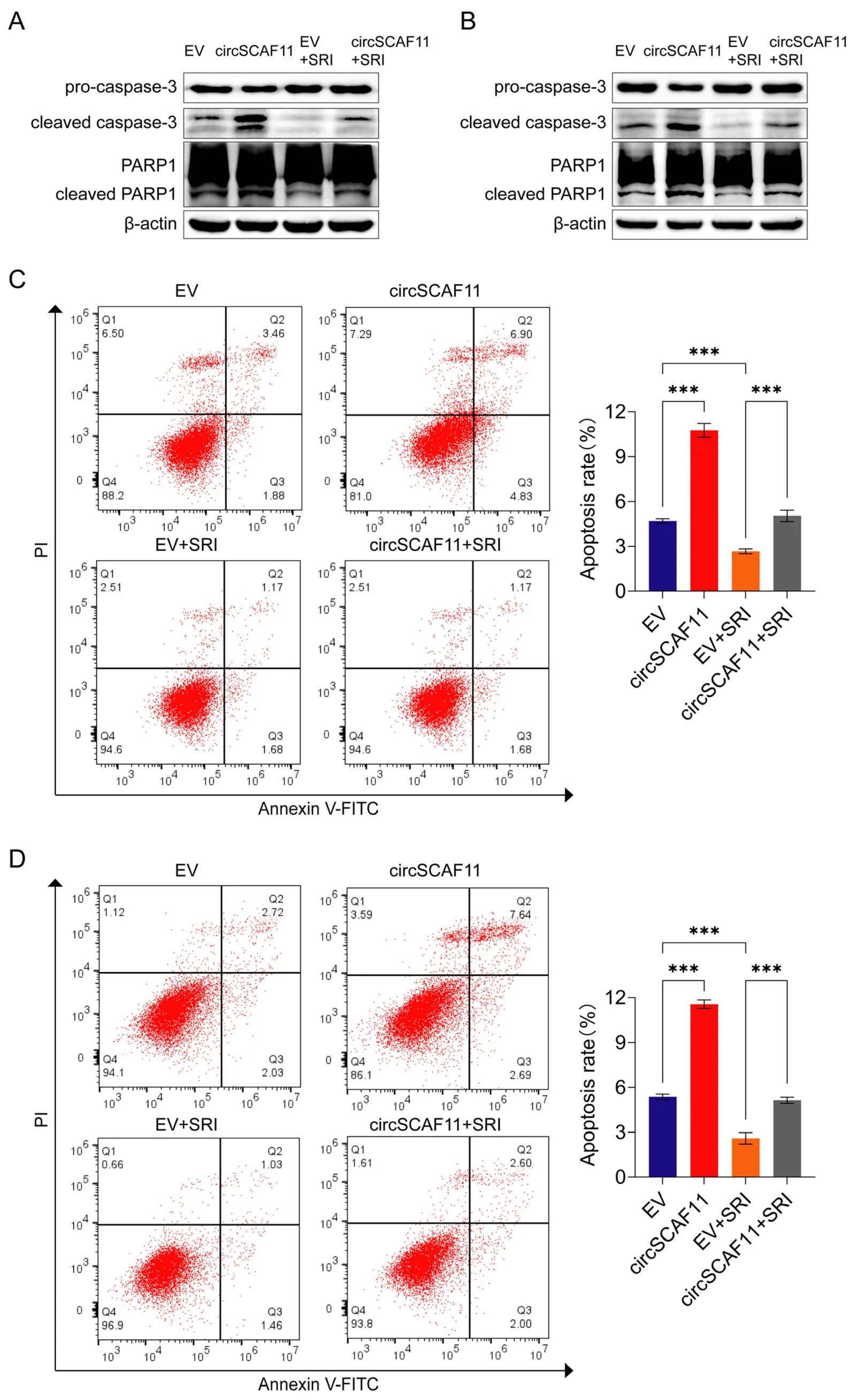
circSCAF11 overexpression promotes CRC cell apoptosis by restraining TGF-β/Smad2/3 signaling. (**A**,**B**). Western blot analysis of the protein expression levels of PARP1, cleaved PARP1, caspase-3, and cleaved caspase-3 in HCT116 (**A**) and HT-29 (**B**) cells under the indicated conditions. β-actin was used as a loading control. (**C**,**D**). Annexin V-FITC/PI double staining followed by flow cytometry analysis of apoptosis in HCT116 (**C**) and HT-29 (**D**) cells. Left panels: representative flow cytometry scatter plots; right panels: quantitative analysis of the percentage of apoptotic cells (early apoptotic cells in Q3 + late apoptotic cells in Q2). The whole blots (uncropped blots) are shown in the Supplementary File. *** *p* < 0.001.

**Figure 6:**
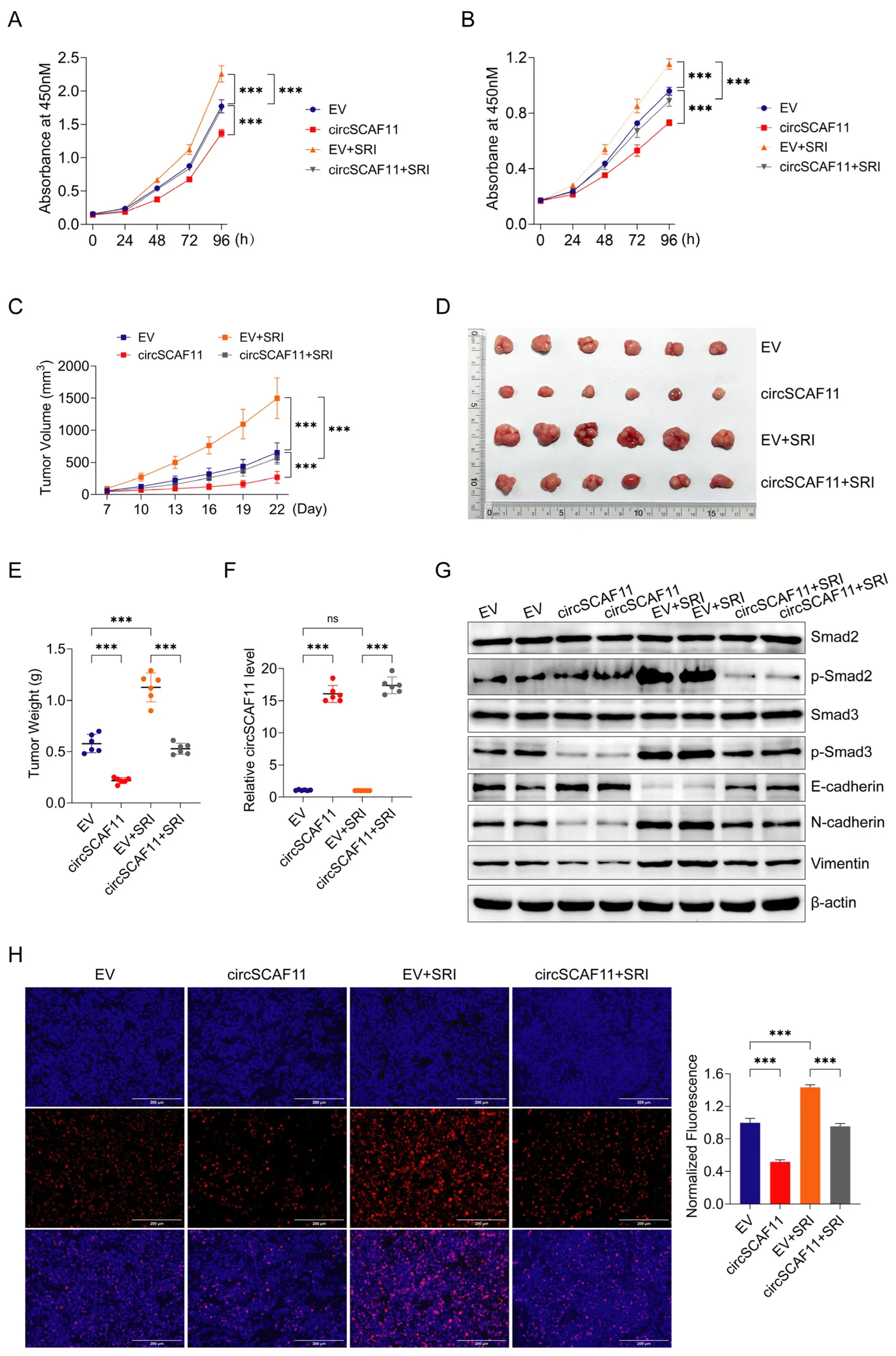
circSCAF11 suppresses CRC tumor growth in vivo. (**A**,**B**). Cell proliferation curves of HCT116 (**A**) and HT-29 (**B**) cells in the EV control group, circSCAF11 overexpression group, EV + SRI group, and circSCAF11 + SRI group over a period of 0–96 h. (**C**). Growth curves of subcutaneous xenograft tumor volumes. Two-way repeated-measures ANOVA was used for statistical analysis. (**D**). Representative images of xenograft tumors excised on day 22 (*n* = 6). (**E**). Statistical analysis of final tumor weights in each group (*n* = 6) was performed using one-way ANOVA. (**F**). RT-qPCR analysis of circSCAF11 expression in xenograft tumors from each group (*n* = 6), with β-actin as a normalization control. (**G**). The protein abundance of Smad2, p-Smad2, Smad3, p-Smad3, E-cadherin, N-cadherin, and Vimentin in xenograft tumor tissues was measured by Western blotting, with β-actin as a loading control. (**H**). Representative images of Ki67 immunofluorescence staining and quantitative analysis of the positive rate. Scale bar: 200 μm. The whole blots (uncropped blots) are shown in the Supplementary File. ns: not significant; *** *p* < 0.001.

## Data Availability

The authors confirm that all the data generated or analyzed during this study are included in this article.

## Supplementary Materials

The following supporting information can be downloaded at: https://www.mdpi.com/article/10.3390/cancers18193128/s1, Figure S1: CircSCAF11 lacks the potential to be translated into proteins or peptides. Figure S2: A higher apoptosis rate is observed in tumor cells overexpressing circSCAF11 in a CRC nude mouse xenograft model. Table S1: Primers for RT-PCR. Table S2: Primers for quantitative Real-time PCR (qPCR). Table S3: Primers for the construction of circSCAF11 overexpression plasmids. Supplementary file: Western blot—Raw blots.

## Abbreviations

CRC	Colorectal cancer
EMT	epithelial–mesenchymal transition
circRNA	circular RNA
qPCR	quantitative real-time PCR
IRES	internal ribosome entry site
cDNA	complementary DNA
gDNA	genomic DNA
EV	empty vector
SRI	SRI-011381
p-Smad2	phosphorylated Smad2
p-Smad3	phosphorylated Smad3
