# Supplementary material for: circSCAF11 Attenuates TGF-β/SMAD2/3 Signaling to Suppress Colorectal Cancer Progression
Source: Cancers (Basel). 2026 Sep 27;18(19):3128. doi: 10.3390/cancers18193128 (PMC13664225; doi:10.3390/cancers18193128)
Supplement: Supplementary file 1 [file cancers-18-03128-s001.zip › Figures and Tables.pdf]

A

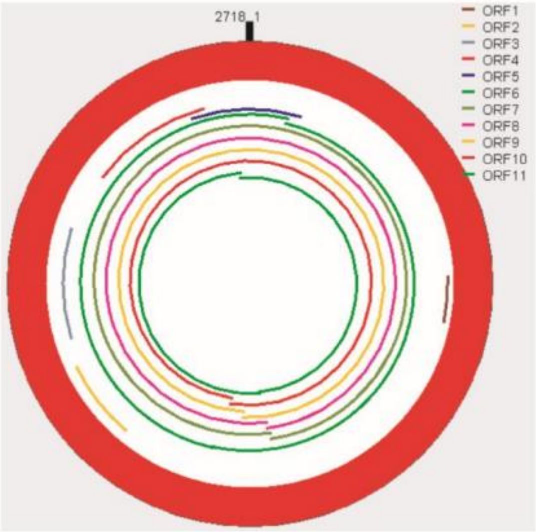

B

Lable	Frame	Start	Stop	Length(nt|aa)	IRES	m^6A High-confidence
ORF1	1	667	771	105|34	None	None
ORF2	1	1669	1854	186|61	None	None
ORF3	1	1915	2175	261|86	None	None
ORF4	1	2314	2610	297|98	None	None
ORF5	2	2582	136	273|90	None	None
ORF6	3	105	104	2718×n|906×n	None	None
ORF7	3	1305	1304	2718×n|906×n	None	None
ORF8	3	1314	1313	2718×n|906×n	None	None
ORF9	3	1389	1388	2718×n|906×n	None	None
ORF10	3	1428	1427	2718×n|906×n	None	None
ORF11	3	2688	2687	2718×n|906×n	None	None

Figure S1. CircSCAF11 lacks the potential to be translated into proteins or peptides.
A. ORF prediction of circSCAF11 using CircPrimer 2.0. **B.** CircPrimer 2.0-based prediction of IRES and m^6A features in circSCAF11.

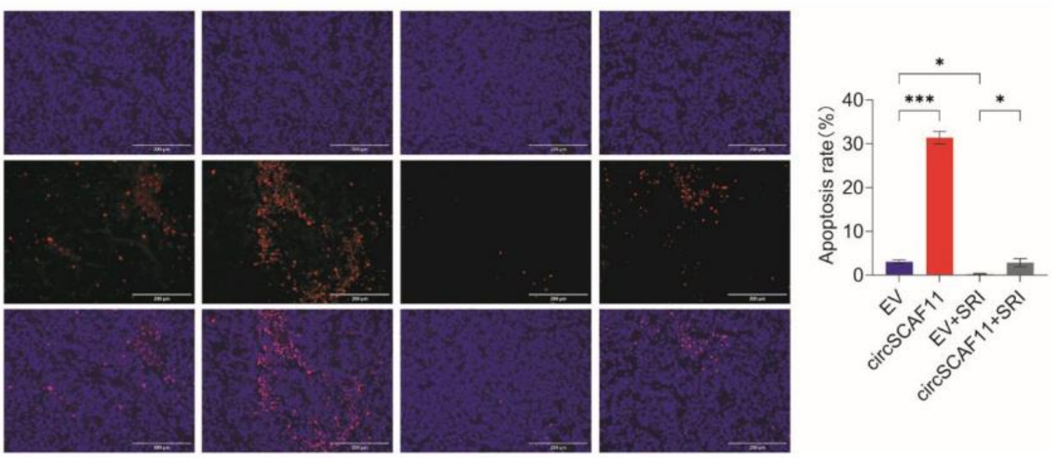

Figure S2: A higher apoptosis rate is observed in tumor cells overexpressing circSCAF11 in a CRC nude mouse xenograft model.

Table S1. Primers for RT-PCR

Oligo name	Sequence (5'-3')
circSCAF11 Divergent primer Forward	TCCACAGTCTGGATGGATGA
circSCAF11 Divergent primer Reverse	TGTCATTGCAGGTTTTCGTC
circSCAF11 Convergent primer Forward	GTACTTCTTGCAAGGGATATGCA
circSCAF11 Convergent primer Reverse	GACTCAGCTGAAGAGGGAACA
β-actin Divergent primer Forward	AAAGGCGAGGCTCTGTGCT
β-actin Divergent primer Reverse	GGGCTTACCTGTACACTGACTTGA
β-actin Convergent primer Forward	AGAAAATCTGGCACCACACC
β-actin Convergent primer Reverse	GGGGTGTTGAAGGTCTCAAA

Table S2. Primers for quantitative Real-time PCR (qPCR)

Oligo name	Sequence (5'-3')
circSCAF11 Forward	TCCACAGTCTGGATGGATGA
circSCAF11 Reverse	TGTCATTGCAGGTTTTCGTC
SCAF11 Forward	CCTCTCATTTCTTCTGTGTTGCC
SCAF11 Reverse	GCAGGTTTTCGTCTTGATCCTC
β-actin Forward	AGAAAATCTGGCACCACACC
β-actin Reverse	GGGGTGTTGAAGGTCTCAAA
GAPDH Forward	ATGTTCGTCATGGGTGTGAA
GAPDH Reverse	GTCTTCTGGGTGGCAGTGAT
U1 Forward	TCCCAGGGCGAGGCTTATCCATT
U1 Reverse	GAACGCAGTCCCCCACTACCACAAAT
TGF-β1 Forward	GCTGAGGTATCGCCAGGAAT
TGF-β1 Reverse	GCTGAGGTATCGCCAGGAAT

TGF-β2 Forward	GCCTGAACAACGGATTGAGC
TGF-β2 Reverse	ATCGAAGGAGAGCCATTCGC
TGF-β3 Forward	CGAGCAGAATTCCGGGTCTT
TGF-β3 Reverse	ATCAAAGGACAGCCACTCGG
TGFBR1 Forward	GGTACTTGGACTCAGCTCTGG
TGFBR1 Reverse	CTCTATGAGCAATGGCTGGCT
TGFBR2 Forward	CCAAGCTCCCCTACCATGAC
TGFBR2 Reverse	GCACTCATCAGAGCTACAGGA
NOG Forward	CACTATCTCCACATCCGCCC
NOG Reverse	CCATGAAGCCTGGGTCGTAG
THBS1 Forward	CTGCAAAAAGGTGTCCTGCC
THBS1 Reverse	TGCCACAGCTCGTAGAACAG
FBN1 Forward	CGGTGTGAGTGCAACAAAGG
FBN1 Reverse	GGCACTGACAGGTGTACGAA
Smad1 Forward	TGAAAACACCAGGCGGCATA
Smad1 Reverse	AGTTGCAGTTCCGACTTTGC
Smad2 Forward	CGCTGGCCTGATCTTCACA
Smad2 Reverse	GTCATCCAGAGGCGGAAGTT
Smad3 Forward	TCTGCGTGAATCCCTACCAC
Smad3 Reverse	TTTTCGGGGATGGAATGGCT
Smad4 Forward	CCATCGTTGTCCACTGAAGGA
Smad4 Reverse	AAGTTGGCAGTGCTGGTAGC
Smad5 Forward	TGTTGGTGGAGAGGTGTATGC

Smad5 Reverse	TGCAGCTGCTGGGAATCTTA
Smad6 Forward	CTCCCTACTCTCGGCTGTCT
Smad6 Reverse	TGGCGTCTGAGAATTCACCC
Smad7 Forward	AGAGGCTGTGTTGCTGTGAA
Smad7 Reverse	GCACAGCATCTGGACAGTCT
Smad9 Forward	GACATTCCAGGCTTCCTCCC
Smad9 Reverse	CACTCGGCATACACCTCTCC

Table S3. Primers for the construction of circSCAF11 overexpression plasmids

Oligo name	Sequence (5'-3')
circSCAF11-Forward	AATATTTCTTCTTTCGAATTCTAATACTTTCAGGTACTTCTT GCAAGGGATATGC
circSCAF11-Reverse	TGGAGTTGTTAGCTAGGATCCAGTTGTTCTTACCCTGTCC AGATGTTTCCTCC
