# Supplementary material for: circSCAF11 Attenuates TGF-β/SMAD2/3 Signaling to Suppress Colorectal Cancer Progression
Source: Cancers (Basel). 2026 Sep 27;18(19):3128. doi: 10.3390/cancers18193128 (PMC13664225; doi:10.3390/cancers18193128)
Supplement: Supplementary file 1 [file cancers-18-03128-s001.zip › Western blot - Raw blots.pdf]

Figure 1F

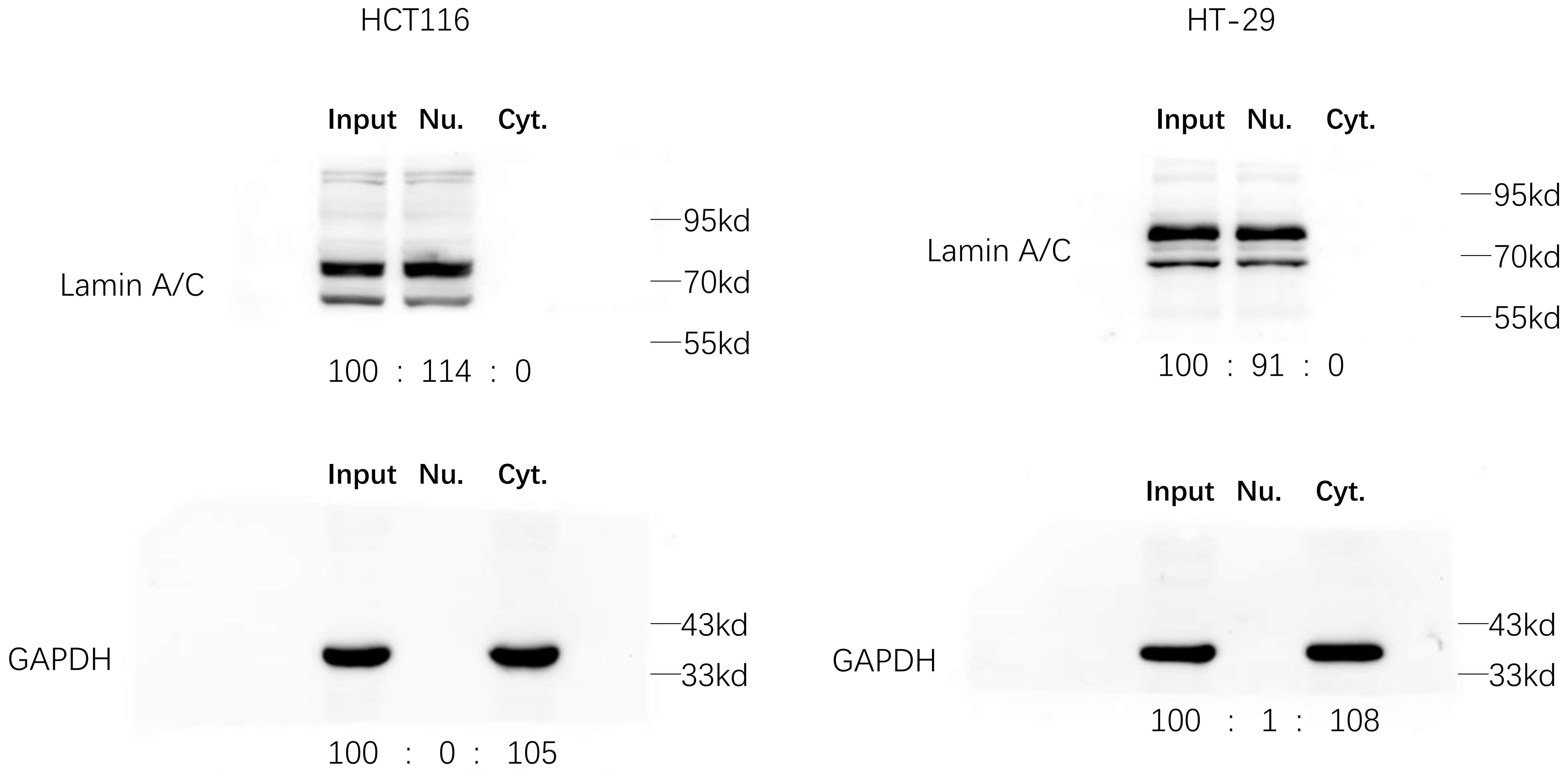

HCT116
HT-29
Input Nu. Cyt.
Lamin A/C
95kd
70kd
55kd
100 : 114 : 0
Input Nu. Cyt.
Lamin A/C
95kd
70kd
55kd
100 : 91 : 0
Input Nu. Cyt.
GAPDH
43kd
33kd
100 : 0 : 105
Input Nu. Cyt.
GAPDH
43kd
33kd
100 : 1 : 108

Figure 3F

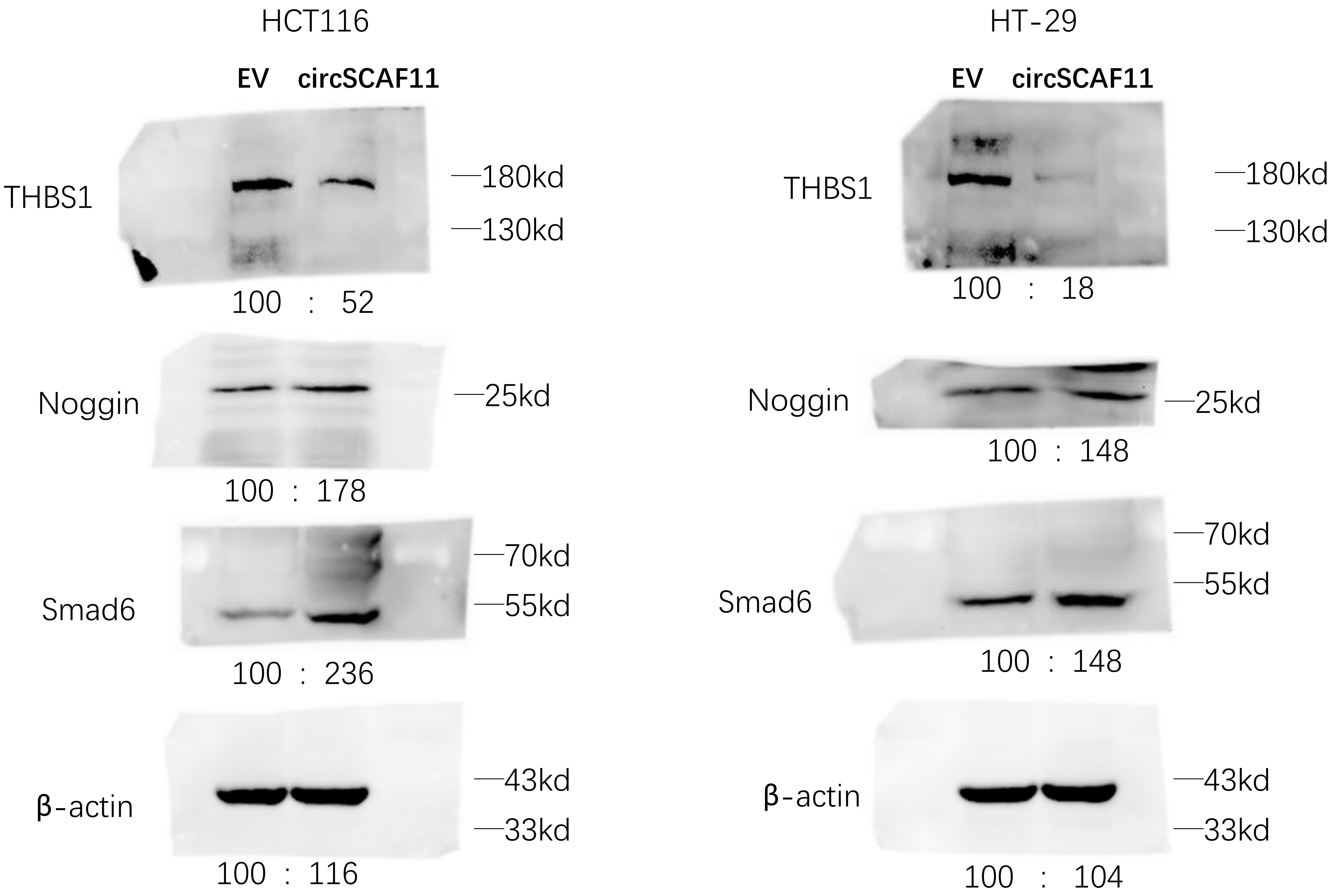
HCT116
EV circSCAF11
THBS1
180kd
130kd
100 : 52
Noggin
25kd
100 : 178
Smad6
70kd
55kd
100 : 236
β-actin
43kd
33kd
100 : 116
HT-29
EV circSCAF11
THBS1
180kd
130kd
100 : 18
Noggin
25kd
100 : 148
Smad6
70kd
55kd
100 : 148
β-actin
43kd
33kd
100 : 104

Figure 4A

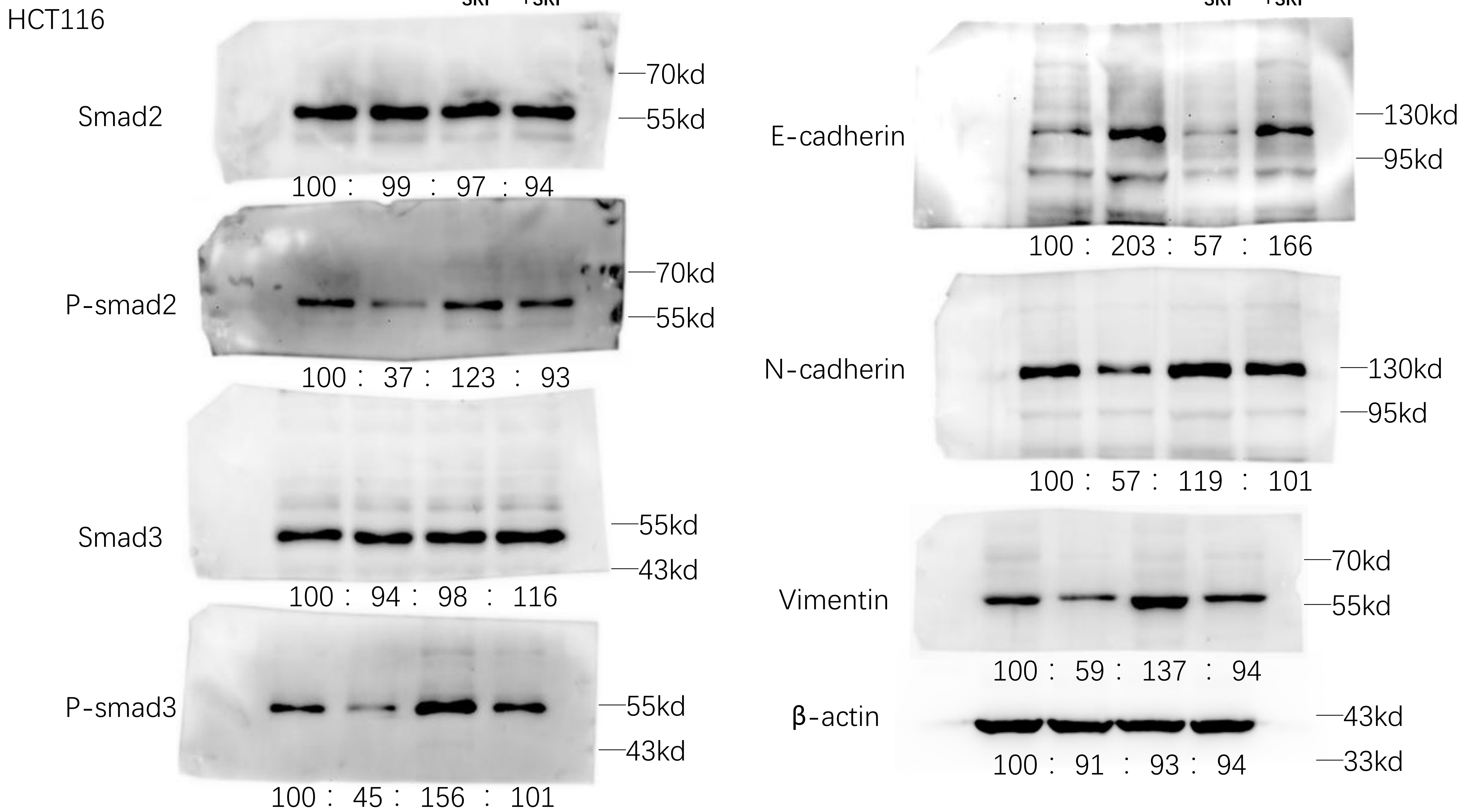
HCT116
EV
circSCAF11
EV+ SRI
circSCAF11 +SRI
Smad2
70kd
55kd
100 : 99 : 97 : 94
P-smad2
70kd
55kd
100 : 37 : 123 : 93
Smad3
55kd
43kd
100 : 94 : 98 : 116
P-smad3
55kd
43kd
100 : 45 : 156 : 101
EV
circSCAF11
EV+ SRI
circSCAF11 +SRI
E-cadherin
130kd
95kd
100 : 203 : 57 : 166
N-cadherin
130kd
95kd
100 : 57 : 119 : 101
Vimentin
70kd
55kd
100 : 59 : 137 : 94
β-actin
43kd
33kd
100 : 91 : 93 : 94

Figure 4B

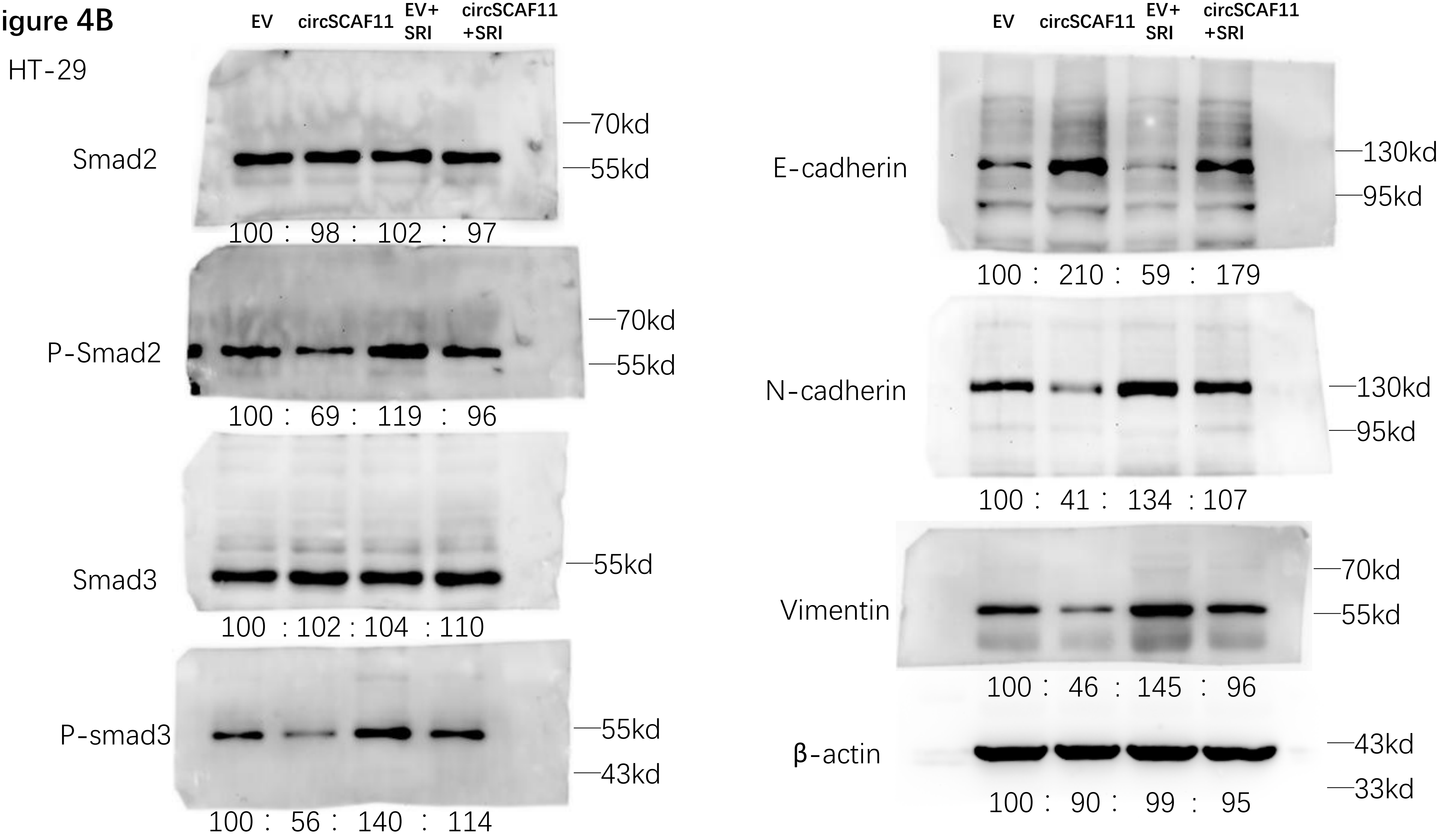
HT-29
EV
circSCAF11
EV+ SRI
circSCAF11 +SRI
Smad2
70kd
55kd
100 : 98 : 102 : 97
P-Smad2
70kd
55kd
100 : 69 : 119 : 96
Smad3
55kd
100 : 102 : 104 : 110
P-smad3
55kd
43kd
100 : 56 : 140 : 114
EV
circSCAF11
EV+ SRI
circSCAF11 +SRI
E-cadherin
130kd
95kd
100 : 210 : 59 : 179
N-cadherin
130kd
95kd
100 : 41 : 134 : 107
Vimentin
70kd
55kd
100 : 46 : 145 : 96
β-actin
43kd
33kd
100 : 90 : 99 : 95

Figure 5A, B

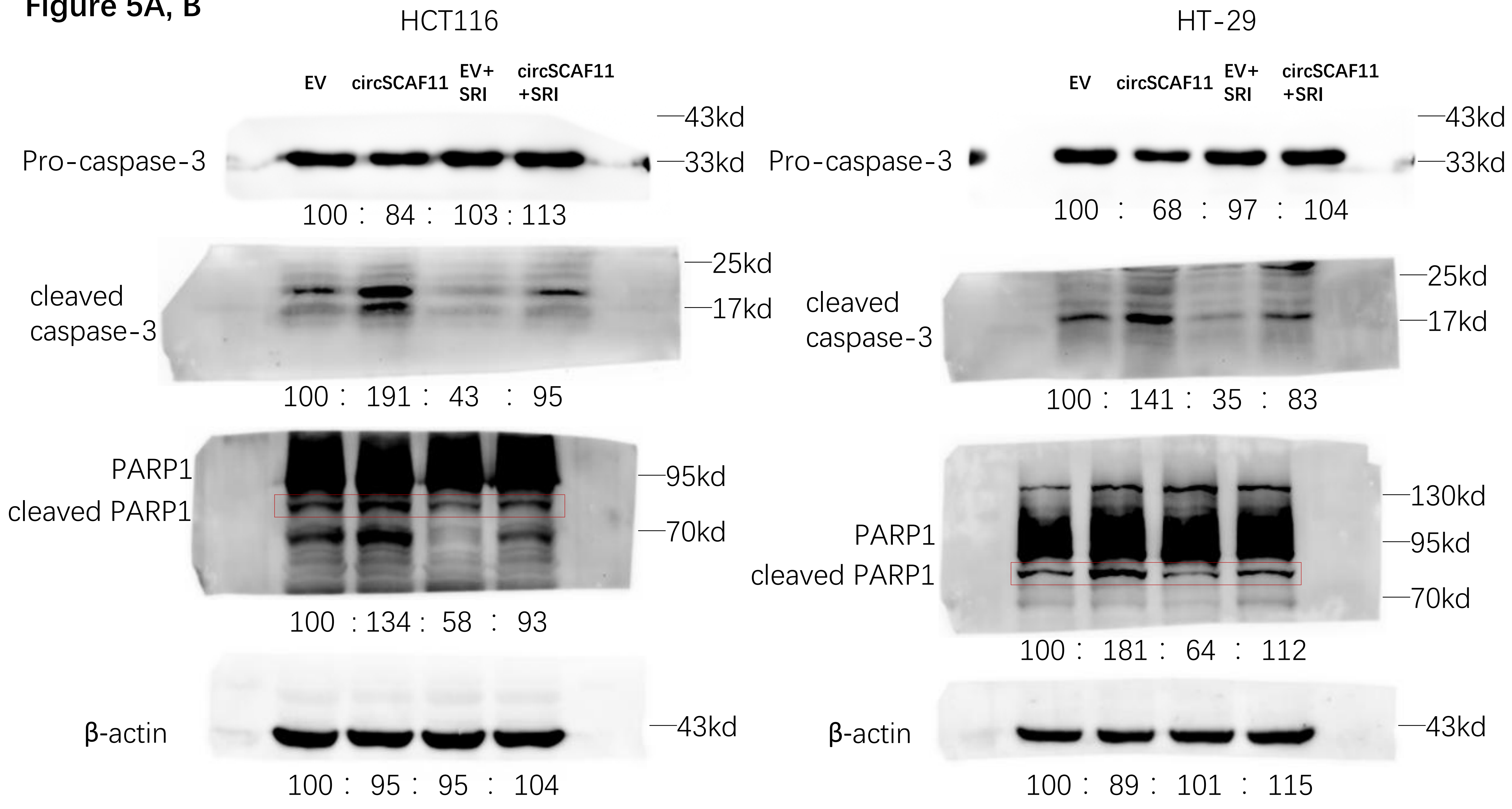

HCT116
EV
circSCAF11
EV+ SRI
circSCAF11 +SRI
43kd
Pro-caspase-3
33kd
100 : 84 : 103 : 113
25kd
cleaved caspase-3
17kd
100 : 191 : 43 : 95
PARP1
95kd
cleaved PARP1
70kd
100 : 134 : 58 : 93
β-actin
43kd
100 : 95 : 95 : 104
HT-29
EV
circSCAF11
EV+ SRI
circSCAF11 +SRI
43kd
Pro-caspase-3
33kd
100 : 68 : 97 : 104
25kd
cleaved caspase-3
17kd
100 : 141 : 35 : 83
130kd
PARP1
95kd
cleaved PARP1
70kd
100 : 181 : 64 : 112
β-actin
43kd
100 : 89 : 101 : 115

Figure 6G

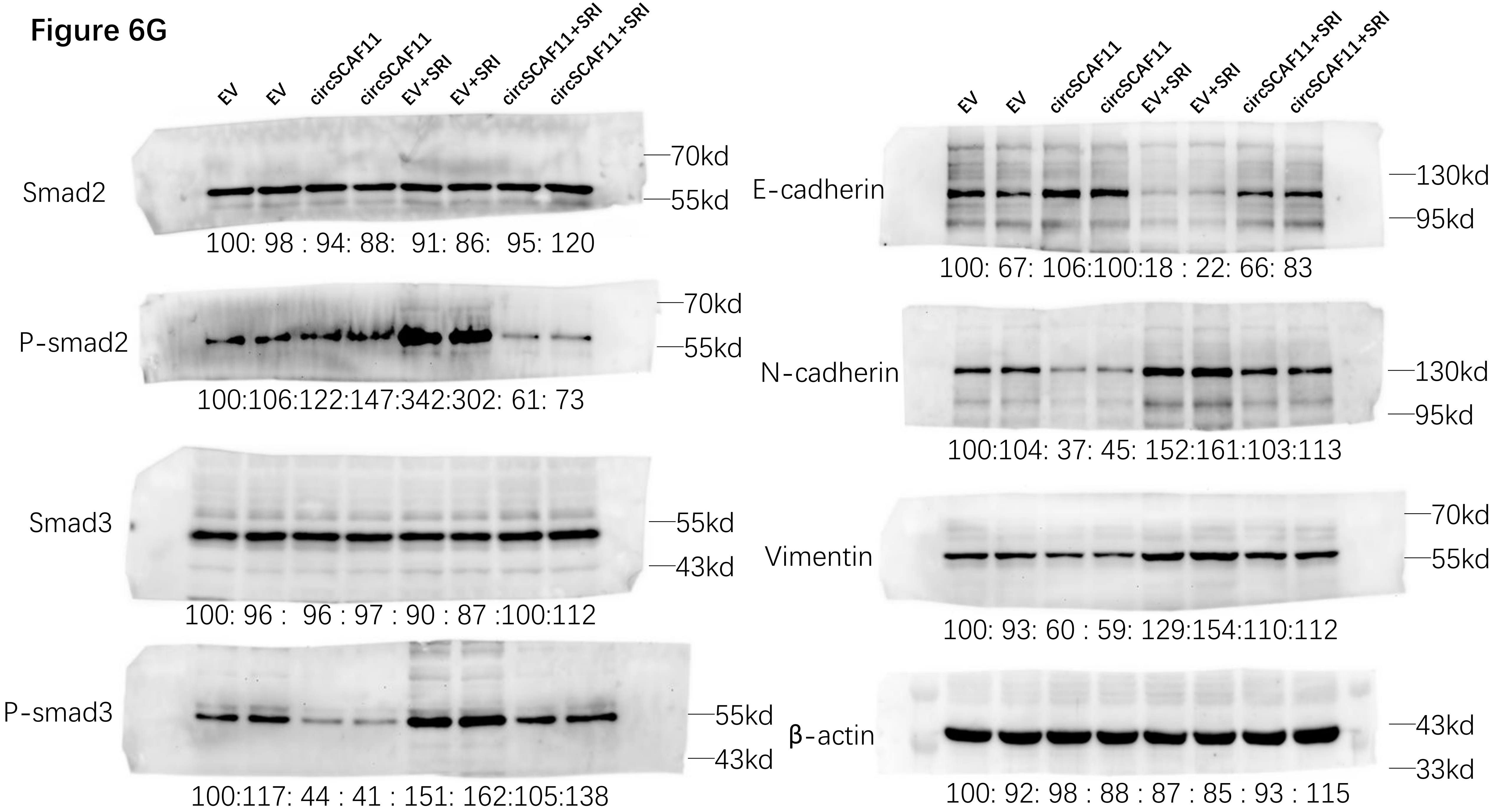
EV
EV
circSCAF11
circSCAF11
EV+SRI
EV+SRI
circSCAF11+SRI
circSCAF11+SRI
Smad2
70kd
55kd
100: 98 : 94: 88: 91: 86: 95: 120
P-smad2
70kd
55kd
100:106:122:147:342:302: 61: 73
Smad3
55kd
43kd
100: 96 : 96 : 97 : 90 : 87 :100:112
P-smad3
55kd
43kd
100:117: 44 : 41 : 151: 162:105:138
E-cadherin
130kd
95kd
100: 67: 106:100:18 : 22: 66: 83
N-cadherin
130kd
95kd
100:104: 37: 45: 152:161:103:113
Vimentin
70kd
55kd
100: 93: 60 : 59: 129:154:110:112
β-actin
43kd
33kd
100: 92: 98 : 88 : 87 : 85 : 93 : 115
